# Dynamics of Polyphenol Biosynthesis by Calli Cultures, Suspension Cultures and Wild Specimens of the Medicinal Plant *Ligaria cuneifolia* (Ruiz & Pav.) Tiegh. (Loranthaceae). Analysis of Their Biological Activity

**DOI:** 10.3390/plants10081713

**Published:** 2021-08-20

**Authors:** María Valeria Ricco, Martín León Bari, Alejandra Vanina Catalano, Paula López, Cecilia Beatriz Dobrecky, Sergio Adrián Teves, Ariana Posadaz, Melina Laguia Becher, Rafael Alejandro Ricco, Marcelo Luis Wagner, María Alejandra Álvarez

**Affiliations:** 1 Centro de Estudios Biomédicos, Básicos, Aplicados y Desarrollo (CEBBAD), Facultad de Ciencias de la Salud, Universidad Maimónides, Hidalgo 775, Ciudad Autónoma de Buenos Aires 1405, Argentina; ricco.mariavaleria@maimonides.edu (M.V.R.); bari.martin@maimonides.edu (M.L.B.); melinalb@gmail.com (M.L.B.); 2 Consejo Nacional de Investigaciones Científicas y Técnicas, Godoy Cruz 2290, Ciudad Autónoma de Buenos Aires 1425, Argentina; 3 Cátedra de Farmacognosia, Facultad de Farmacia y Bioquímica, Universidad de Buenos Aires, Junín 956, Ciudad Autónoma de Buenos Aires 1113, Argentina; alejandracatalano@gmail.com (A.V.C.); plopez@ffyb.uba.ar (P.L.); 4 CONICET, Instituto de la Química y Metabolismo del Fármaco (IQUIMEFA), Junín 956, Ciudad Autónoma de Buenos Aires 1113, Argentina; 5 Cátedra de Farmacobotánica, Departamento de Farmacología, Facultad de Farmacia y Bioquímica, Universidad de Buenos Aires, Junín 956, Ciudad Autónoma de Buenos Aires 1113, Argentina; cecilia.dobrecky@gmail.com (C.B.D.); raricco@gmail.om (R.A.R.); mlwagner@ffyb.uba.ar (M.L.W.); 6 Departamento de Tecnología Farmacéutica, Cátedra de Tecnología Farmacéutica I, Facultad de Farmacia y Bioquímica, Universidad de Buenos Aires, Junín 956, Ciudad Autónoma de Buenos Aires 1113, Argentina; 7 Cátedra de Microbiología, Facultad de Farmacia y Bioquímica, Universidad de Buenos Aires, Junín 956, Ciudad Autónoma de Buenos Aires 1113, Argentina; steves@proanalisis.com.ar; 8 Proanálisis S.A, Av. San Martin 2355, Ciudad de Buenos Aires 1416, Argentina; 9 Facultad de Turismo y Urbanismo, Universidad Nacional de San Luis, Av. del Libertador s/n, Barranca Colorada, Villa de Merlo, San Luis 5881, Argentina; arianaposadaz@yahoo.com.ar

**Keywords:** antioxidant capacity, condensed tannins, dedifferentiated cultures, flavonoids, hemiparasite, hydroxycinnamic acids, mistletoe, phenolics, secondary metabolites

## Abstract

*Ligaria cuneifolia* (R. et P.) Tiegh. (Loranthaceae) is a South American hemiparasitic species with antioxidant, antitumoral, antimicrobial, and antilipidemic activities attributed to its polyphenolic content. We studied the polyphenolic pattern of *L. cuneifolia* during different phenological stages: flowering, fruiting, and post-fruiting. The highest total phenolic content was found in stems at post-fruiting (214 ± 12.1 mg gallic acid eq·g^−1^ DW) and fruiting (209 ± 13.7 mg gallic acid eq·g^−1^ DW), followed by post-fruiting leaves (207 ± 17.5 mg gallic acid eq·g^−1^ DW). Flavonoids accumulated at higher levels in leaves and hydroxycinnamic acids in leaves at flowering and post-fruiting. The polyphenolic pattern was similar between organs from wild plants and in vitro cultures, although at a significantly lower level in the latter ones. The performance of calli growing under a 16 h photoperiod in a modified White medium with 1-naphthalene acetic acid (2.50 μM) and Kinetin (9.20 μM) was better than in the dark. When calli grew in media only with auxins (IAA, NAA, and 2,4-D, all at 2.50 µM concentration), its growth and polyphenolic content improved. Cell suspensions with 2.50 µM NAA and 9.20 µM KIN grew slowly and produced very small amounts of polyphenols. As for the antioxidant activity, it was detected in all samples (approximately 1000 µmol trolox eq·g^−1^ DW) except fruits, where a lower value was found (328 µmol trolox eq·g^−1^ DW). In vitro cultures have the lowest antioxidant activity when compared to methanolic extracts from organs of wild specimens. Finally, antimutagenic or mutagenic activity in wild plants and in vitro culture extracts was not detected by the Ames test.

## 1. Introduction

Mistletoes are plant species from the Loranthaceae and Santalaceae families, spread worldwide. Among the approximately 1000 Loranthaceae species, 300 are endemic to America. The genus *Ligaria* is represented by *L. teretifolia* (Rizzini) Kuijt, endemic to Brazil, and *L. cuneifolia* (Ruiz & Pav.) Tiegh, from Uruguay, Brazil, Perú, Chile, and Argentina [1,2]. *L. cuneifolia* is a hemiparasite species that, due to its morphological similarities and growth behavior, was used as a substitute for the European mistletoe (*Viscum album* L., Santalaceae) by the first European immigrants [3]. Ethnobotanical studies have reported *L. cuneifolia* use as antihemorrhagic, abortive, emmenagogue, and oxytocic and against cephalgia, gastralgias, sore throat, and hypothermia [2]. On the other hand, pharmacological studies have demonstrated that *L. cuneifolia* extracts decreased cholesterol and lipid blood levels in rats [4,5], had antitumoral activity [6], produced a reduction in cell proliferation in murine lymphoma [7], had a bactericidal effect against phytopathogens and clinical pathogens [8], and displayed a strong in vitro antioxidant activity [9,10,11]. Therefore, *L. cuneifolia* use for the treatment of cardiovascular diseases and cancer is a promising alternative [12,13]. Phytochemical studies have identified several compounds potentially responsible for the above-mentioned activities. The flavonol quercetin (QE) was identified in *L. cuneifolia* specimens growing on different hosts and coming from different regions. QE could be found free or as a 3-*O*-glycoside derivative with glucose, xylose, rhamnose, or arabinose. Leucoanthocyanidins, catechin-4-ß-ol, and proanthocyanidins (PA) as polymers, oligomers, and dimers that produced cyanidin after hydrolysis were also reported [14]. Dobrecky [15] has also identified QE-3-*O*-(2″-*O*-galloyl) rhamnoside, QE-3-*O*-(3″-*O*-galloyl) rhamnoside, QE-3-*O*-(2″ galloyl)-arabinofuranoside, and QE-3-*O*-(2″-*O*-galloyl)-arabinopyranoside. Production of secondary metabolites, including plant polyphenolics, depends on numerous factors, such as growth conditions (light, temperature, altitude, nutrient availability), plant phenological stage, and organ. Changes in the polyphenolic content are related to variations in the expression of the genes encoding the activity of enzymes involved in their biosynthesis [16]. Consequently, the analysis of polyphenolic compounds in different phenological stages and organs is relevant to increase the knowledge on polyphenol dynamics in *L. cuneifolia* and to harvest it at the highest levels of bioactive compounds. In this work, we studied the dynamics of polyphenolic production in different organs of *L. cuneifolia* wild specimens in different phenological stages.

On the other hand, the establishment of *L. cuneifolia* in vitro cultures as a source of plant material appears as an environmentally friendly strategy to avoid the excessive exploitation of the species. In addition, these cultures have the advantage of providing plant material of uniform quality produced under controlled environmental conditions free from pests and diseases. There are only a few reports about the establishment of in vitro cultures from Loranthaceae species [17,18]. We have previously determined the conditions to initiate in vitro calli cultures from *L. cuneifolia* on White medium with 500 mg L^−1^ casein hydrolysate, 100 mg L^−1^ myo-inositol, B5 vitamins, 4% (*w*/*v*) sucrose, 2.50 µM NAA, and 9.20 µM KIN as plant growth regulators (PGRs) and a 16 h photoperiod [19]. The behavior of most plant cultures depends on the quality, intensity, and duration of the light period, since the activity of many of the enzymes involved in the biosynthetic pathways of metabolites is influenced by light [20]. Therefore, in the present work, we continued the study of in vitro calli induction and growth behavior, analyzing the influence of two different photoperiods. Once the calli are formed, it is necessary to improve their growth and plant metabolite production. The influence of PGRs on the production of secondary metabolites in in vitro cultures of different plant species is well known [21,22,23]. Accordingly, in this study, we tested different PGRs on calli growth and polyphenol yield over time. In addition, we studied the influence of inoculum size on cell suspension culture initiation, a key growth variable [21]. We measured their polyphenol production over time as well.

Finally, we tested the antioxidant, mutagenic, and antimutagenic activities of wild specimens and in vitro culture extracts.

## 2. Results

### 2.1. Dynamics of Polyphenols in Different Organs and Phenological Stages of Wild Plants

#### 2.1.1. Spectrophotometric Analysis

We found significant differences in the total phenolic content (TPC; *p* < 0.05) among organs (leaves, stems, flowers, and fruits) in the three studied phenological stages (fruiting, post-fruiting, and flowering). The highest values corresponded to stems at post-fruiting (214 ± 12.1 mg GA eq·g^−1^ DW) and fruiting (209 ± 13.7 mg GA eq·g^−1^ DW), followed by post-fruiting leaves (207 ± 17.5 mg gallic acid eq·g^−1^ DW). The lowest values were found in fruits (81.7 ± 0.00 mg GA eq·g^−1^ DW; Table 1 and Table 2).

**Figure 1:**
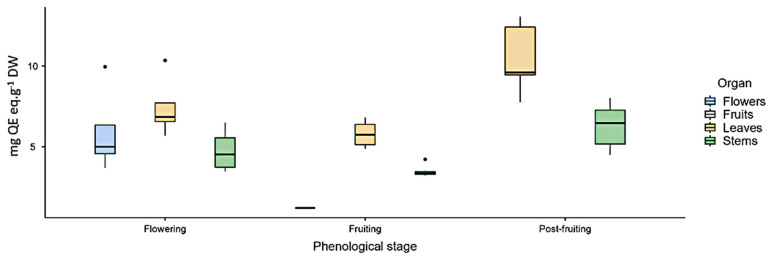
Total flavonoid content (mg quercetin eq·g^−1^ DW) measured by UV spectrophotometry in *Ligaria cuneifolia*’s methanolic extracts of leaves, stems, flowers, and fruits, harvested in different phenological stages (flowering, fruiting, and post-fruiting).

As for flavonoids (FL), the highest content was found in leaves in the three analyzed phenological stages (Figure 1).

Regarding hydroxycinnamic acids (HCA), significant differences (*p* < 0.05) were found between leaves and stems; however, no significant differences (*p* > 0.05) were found among phenological stages. The highest values corresponded to leaves harvested at the flowering (3.02 ± 0.62 mg CA eq·g^−1^ DW) and post-fruiting stages (3.06 ± 0.9 mg CA eq·g^−1^ DW; Table 3).

No significant differences in proanthocyanidin (PA) content were found among the different organs and phenological stages (*p* > 0.05; Table 4).

#### 2.1.2. Thin-Layer Chromatography (TLC) Analysis

**Figure 2:**
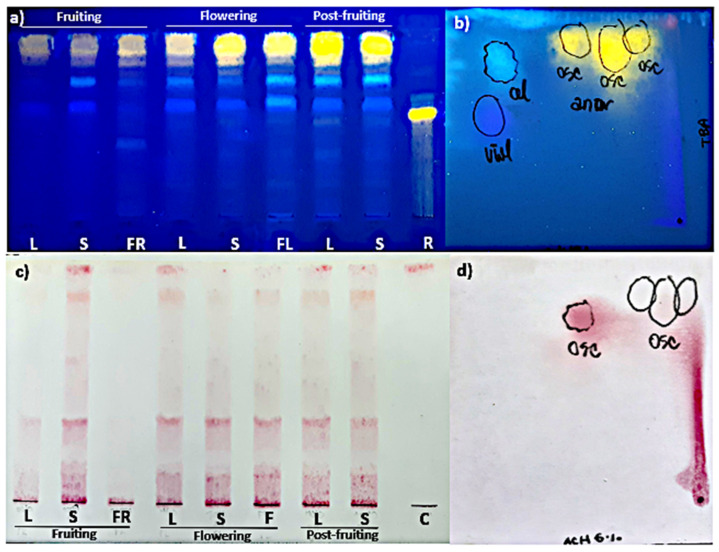
Mono-dimensional (**a**,**c**) and bi-dimensional (**b**,**d**) analysis of methanolic extracts from *Ligaria cuneifolia* organs harvested in different phenological stages. (**a**,**c**) Methanolic extracts of L: leaves; S: stems; FR: fruits; F: flowers; standards: R (rutin) and C (catechin); (**b**,**d**) methanolic extracts of leaves and stems from wild specimens harvested in the post-fruiting stage (equal parts mix). Spray reagents: (**a**,**b**) AEDBE: 2-aminoethyl diphenyl borate ester, Sigma-Aldrich. Wavelength: 366 nm; (**c**,**d**) ethanolic solution of 5% (*v*/*v*) vanillin/hydrochloric acid.

The TLC analysis showed that the pattern of FL and HCA was similar in all organs and phenological stages studied (Figure 2a). As for their content, the highest polyphenolic values corresponded to the post-fruiting stage. The PA (+) catechin was detected in all organs and phenological stages studied (Figure 2c). This finding was confirmed by bi-dimensional TLC where a similar pattern of flavonoids, HCA, and PA was found in leaves and stems harvested in the post-fruiting stage (Figure 2b,d).

#### 2.1.3. HPLC-UV Analysis

**Figure 3:**
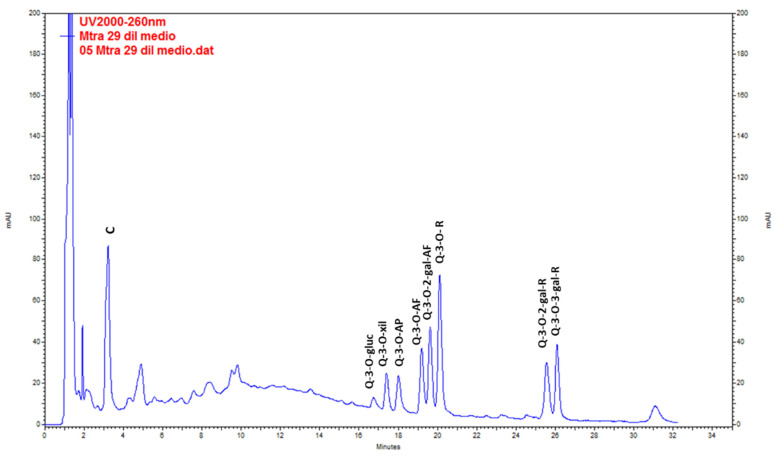
Chromatogram resulting from the analysis by HPLC-UV of the methanolic extract of *Ligaria cuneifolia* stems from specimens harvested in the post-fruiting stage. C (Catechin), Q-3-*O*-gluc (Q-3-*O*-glucoside), Q-3-*O*-xyl (Q-3-*O*-xyloside), Q-3-*O*-AP (Q-3-*O*-arabinopyranoside), Q-3-*O*-AF (Q-3-*O*-arabinofuranoside), Q-3-*O*-2-gal-AF (Q-3-*O*-2-galloyl-arabinofuranoside), Q-3-*O*-R (Q-3-*O*-rhamnoside), Q-3-*O*-2-gal-R (Q-3-*O*-2-galloyl-rhamnoside) and Q-3-*O*-3-gal-R (Q-3-*O*-3-galloyl-rhamnoside).

Catechin was the main compound found in wild plants. QE glycosides (QE-3-*O*-glycoside, QE-3-*O*-xyloside, QE-3-*O*-arabinopyranoside, QE-3-*O*-arabinofuranoside, QE-3-*O*-rhamnoside, QE-3-*O*-2-galloyl-arabinofuranoside, QE-3-*O*-2-galloyl-rhamnoside and QE-3-*O*-3-galloyl-rhamnoside) were found at lower levels (Figure 3, Table 5). The highest content of total metabolites was found in the extracts from stems (20.44 ± 0.36 mg g^−1^ DW), followed by leaves (19.64 ± 0.24 mg g^−1^ DW) in the post-fruiting stage. The lowest values corresponded to fruits (4.32 ± 0.04 mg g^−1^ DW).

#### 2.1.4. Histochemical Analysis

**Figure 4:**
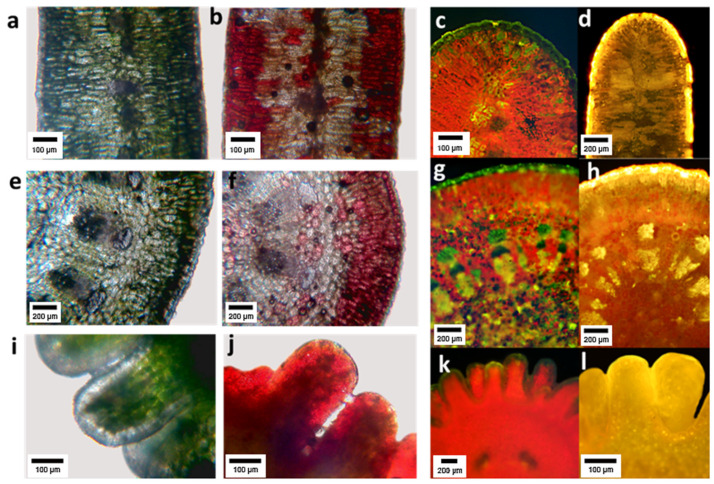
Cross sections of *Ligaria cuneifolia* leaf (**a**–**d**), stem (**e**–**h**) and embryo (**i**–**l**). Subfigures (**a**,**e**,**i**) correspond to cross sections of the three organs prior to reaction with vanillin–hydrochloric acid, observed under a bright-field microscope; the subfigures (**b**,**f**,**j**) correspond to the same sections, but after the reaction with the vanillin–hydrochloric reagent in which a characteristic red coloration typical of proanthocyanidins is observed. Subfigures (**c**,**g**,**k**) are cross sections of the three organs prior to reaction with AEDBE observed under a fluorescence microscope; subfigures (**d**,**h**,**i**) correspond to the same sections, but after reaction with AEDBE in which the presence of flavonoids is revealed by a characteristic yellow coloration.

**Figure 5:**
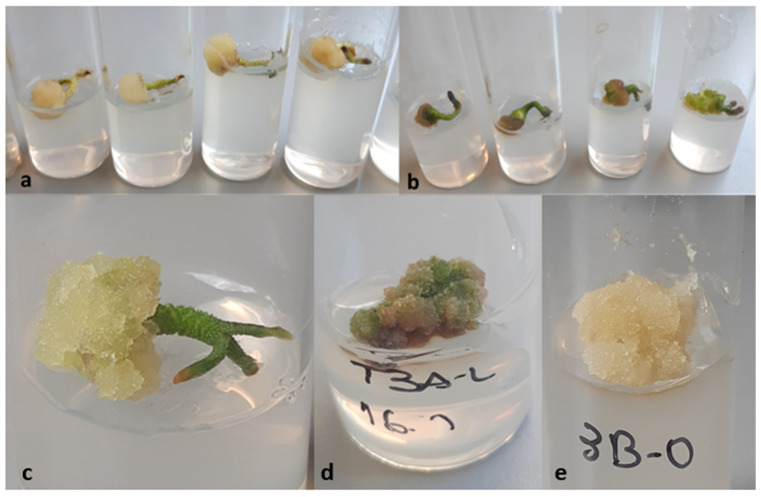
Aspect of calli induced from embryos under continuous darkness (**a**,**c**,**e**) and under a 16 h light photoperiod (**b**,**d**). Note that calli were formed on the extreme of the hypocotyl in all cases (**a**–**c**). Culture media: CWM with 2.5 μM NAA: 9.2 μM KIN.

Histochemical analysis showed that flavonoids accumulated in the epidermis and in the first parenchyma layers of leaves, and with less intensity in the deeper layers of tissue (Figure 4c,d). PA were found in the epidermis, the assimilating parenchyma and vaguely in the central spongy parenchyma (Figure 4a,b). In the primary stems, FL accumulated mainly in the epidermis and first layers of parenchyma (Figure 4g,h), PA in the epidermis, the primary cortex and at lower concentration in the stem pith (Figure 4e,y,f). In the embryo, FL were found in the external tissues (Figure 5k,l), and PA in the epidermis and at a high concentration in parenchyma (Figure 4i,j).

### 2.2. In Vitro Culture Initiation, Growth Kinetics, and Polyphenolic Content under Different Culture Conditions

#### 2.2.1. Light Influence on Calli Induction, Growth and Polyphenolic Content

**Figure 6:**
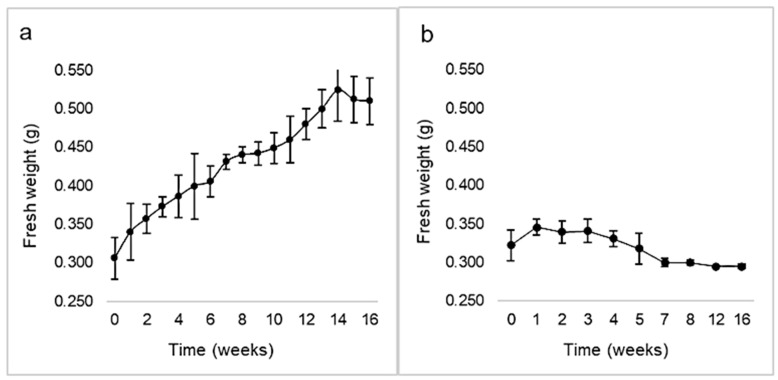
Growth kinetics of *Ligaria cuneifolia* calli initiated under a 16 h photoperiod (**a**) and in darkness (**b**), expressed as fresh biomass (g) over time (weeks). Calli were grown in CWM with 2.5 μM NAA and 9.2 μM KIN.

Calli induction rate was 84.65 ± 9.23% in darkness, while it was 71.19 ± 3.70% with a 16 h photoperiod. No correlation between both variables was demonstrated by chi-square and Fisher’s tests (*p* > 0.05). However, the calli morphology differed, being green and compact under light, or whitish and friable in the dark (Figure 5). The kinetic parameters corresponding to calli growing under illumination were GI = 1.17, µ = 0.01, and d_t_ = 62.7 (Figure 6). Maximal biomass (524 ± 40 mg FW) was achieved at week 14. The cell growth curve did not display a lag phase, showed a very slow exponential phase, and entered the stationary phase by week 14. On the other hand, after one week in darkness calli arrested their growth (maximal biomass: 346 ± 10 mg FW) and by the 3rd week entered the death phase.

**Figure 7:**
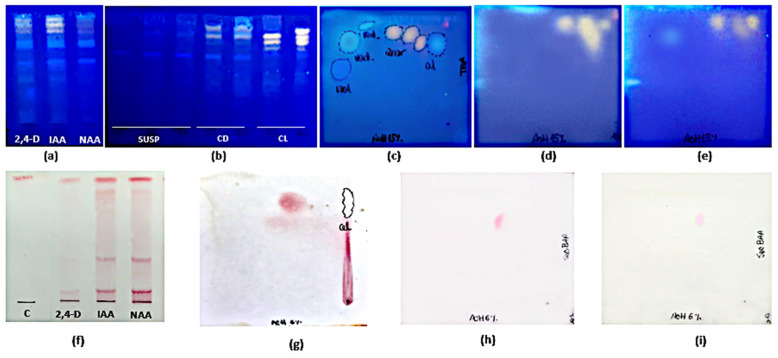
Mono-dimensional (**a**,**b**,**f**) and bi-dimensional (**c**–**e**,**g**–**i**) TLC analysis of methanolic extracts of *Ligaria cuneifolia* in vitro cultures. (**a**,**f**) Calli grown in CWM with different PGRs (IAA, NAA, and 2,4-D) at a concentration of 2.5 µM; (**b**) Cell suspensions (SUSP), calli grown under 16 h photoperiod (CL) and in darkness (CD) in CWM with 2.50 µM NAA and 9.20 µM KIN; (**c**,**g**) calli grown in White medium with 2,5 µM NAA harvested at 6th week of culture; (**d**,**h**) calli grown in CWM under 16 h photoperiod harvested at the 2nd week of culture; (**e**,**i**) calli grown in CWM under continuous darkness harvested at the 2nd week of culture. Spray reagents: (**a**–**e**): AEDBE: 2-aminoethyl diphenyl borate ester, Sigma-Aldrich, wavelength: 366 nm; (**f**–**i**): ethanolic solution of 5% (*v*/*v*) vanillin/hydrochloric acid. 2,4-D: 2,4-dichlorophenoxyacetic acid; IAA: indoleacetic acid; NAA: 1-naphthaleneacetic acid; C: catechin.

As for TPC, FL, and HCA contents, significant differences (*p* < 0.001) were found between cultures maintained under illumination or in darkness. Maximum yields for calli grown under light were 7.84 ± 0.01 mg GA eq·g^−1^ DW at 4th week, 1.86 ± 0.16 mg QE eq·g^−1^ DW and 0.26 ± 0.07 mg CA eq·g^−1^ DW at the 2nd week of culture, respectively. For calli grown in darkness, maximum yields were 6.62 ± 0.01 mg GA eq·g^−1^ DW and 1.11 ± 0.01 mg QE eq·g^−1^ DW at the 2nd week, and 0.12 ± 0.06 mg CA eq·g^−1^ DW at the 1st week of culture, respectively. PA was not detected by spectrophotometry in any case. On the other hand, TLC revealed more intense spots in extracts from calli grown under 16 h photoperiod than in darkness. The PA(+) catechin was only detected by this method (Figure 7b,d,e,h,i). HPLC-UV analysis showed that the total amount of polyphenols in calli was 2-fold higher in light (0.87 ± 0.08 mg g^−1^) than in darkness (0.40 ± 0.02 mg g^−1^) methanolic extracts (Table 5).

#### 2.2.2. Influence of PGRs and Inoculum Size on Growth Kinetics and Polyphenolic Content

**Figure 8:**
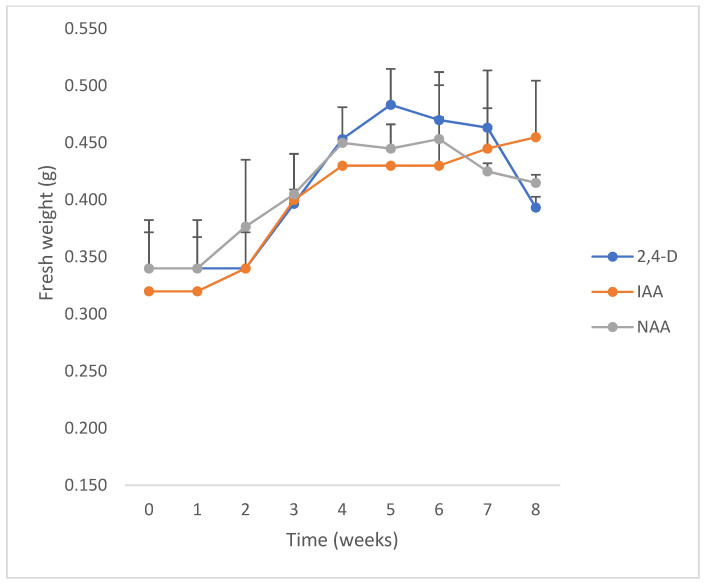
Growth kinetics of *Ligaria cuneifolia* calli grown in a semi-solid medium (CWM), with different auxins (IAA, NAA or 2,4-D at 2.5 µM), initiated from “high” inocula (250 to 500 mg FW). Photoperiod 16 h, temperature 24 ± 2 °C.

All PGRs at the lowest concentration (2.5 µM) with inoculum sizes between 250 and 500 mg FW per tube were effective in inducing growth (Figure 8). On the other hand, none of them were effective at the higher concentration of auxins (5 and 10 µM) or inoculum sizes lower than 250 mg FW per tube. The cell growth curves showed that the performance of calli growing in culture media with IAA and 2,4-D as PGR was better than with NAA. No significant differences were observed between the GI and growth specific rate (µ) from cultures growing in media with IAA (1.60 and 0.02 d^−1^, respectively) or 2,4-D (1.57 and 0.02 d^−1^, respectively), while GI and µ were lower with NAA (1.28 and 0.01 d^−1^, respectively). The doubling times (d_t_) of calli in media with 2.5 µM 2,4-D, IAA, or NAA as PGR were 30.27 d, 42.27 d and 68.35 d, and the maximal biomass was 513 ± 73 mg FW at the 7th week, 455 ± 49 mg FW at the 8th week, and 453 ± 47 mg FW at the 6th week, respectively.

**Figure 9:**
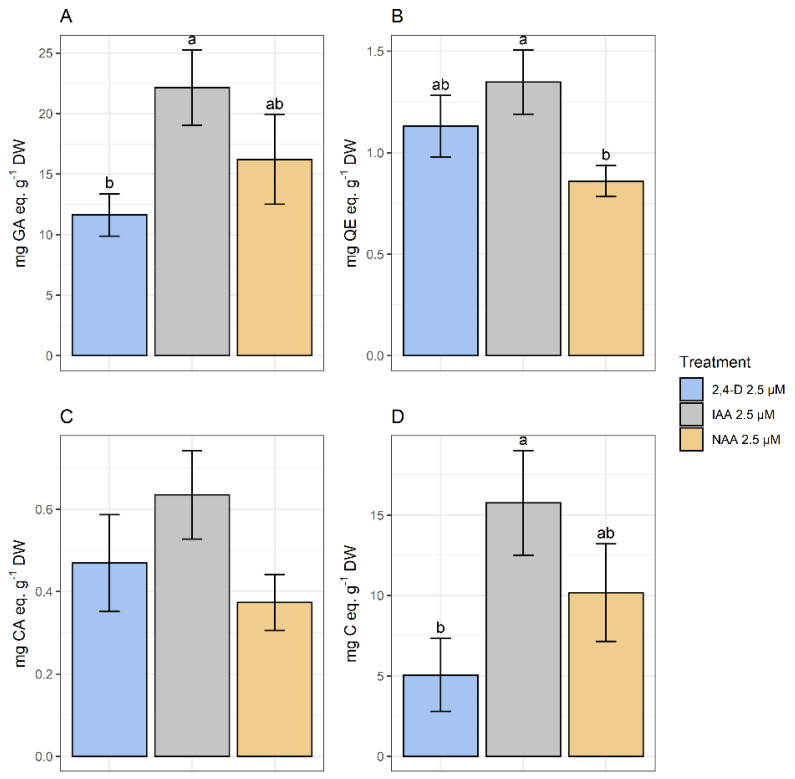
Polyphenolic content of methanolic extracts of calli grown in CWM with different auxins at 2.5 µM. Results are presented as the mean with standard error bars. (**A**) Total phenolics expressed as mg GA eq·g^−1^ DW; (**B**) flavonoids expressed as mg QE eq·g^−1^ DW; (**C**) total hydroxycinnamic acids expressed as mg CA eq·g^−1^ DW; (**D**) total proanthocyanidins expressed as mg C eq·g^−1^ DW. 2,4-D: 2,4-dichlorophenoxyacetic acid; IAA: indoleacetic acid; NAA: naphthaleneacetic acid; GA: gallic acid; QE: quercetin; CA: chlorogenic acid; C: catechin; DW: dry weight. Letters above the bars indicate same groups (*p* < 0.05) according to Tukey’s test (plots **A**,**D**) and Games–Howell test (plot **B**). In plot C, non-significant differences were detected by ANOVA (*p* = 0.174).

The spectrophotometric analysis has determined that the higher TPC corresponded to treatments with IAA and NAA. As for FL, the higher amounts corresponded to calli growing in media with IAA and 2,4-D. No significant differences were found in HCA content among treatments. Finally, the highest amount of PA corresponded to calli growing in the presence of IAA and NAA (Figure 9).

**Figure 10:**
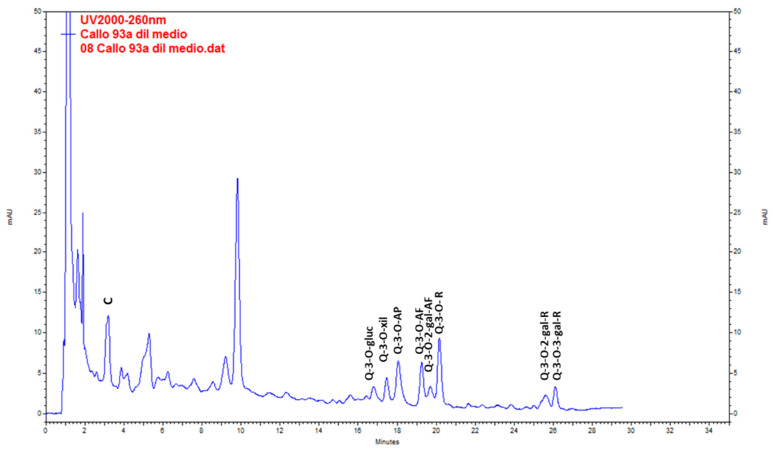
Chromatogram resulting from the HPLC-UV analysis of the methanolic extract of *Ligaria cuneifolia* calli grown in a semi-solid medium (CWM plus 2.5 µM IAA) and harvested at week 6. C (Catechin), Q-3-*O*-gluc (Q-3-*O*-glucoside), Q-3-*O*-xyl (Q-3-*O*-xyloside), Q-3-*O*-AP (Q-3-*O*-arabinopyranoside), Q-3-O-AF (Q- 3-O-arabinofuranoside), Q-3-*O*-2-gal-AF (Q-3-*O*-2-galloyl-arabinofuranoside), Q-3-*O*-R (Q-3-*O*-rhamnoside), Q-3-*O*-2-gal-R (Q-3-*O*-2-galloyl-rhamnoside) and Q-3-*O*-3-gal-R (Q-3-*O*-3-galloyl-rhamnoside).

Mono-dimensional and bi-dimensional TLC confirmed that calli have the same pattern of polyphenolic compounds (Figure 7a,c) as the plant but at a lower concentration. The PA(+) catechin was detected (Figure 7f,g). HPLC-UV analysis (Figure 10) showed that, as in the adult plant, catechin was the main compound in calli, but the amounts were 10- to 20-fold lower (Table 5).

#### 2.2.3. Establishment of Cell Suspension Cultures. Growth Kinetics and Polyphenolic Content

**Figure 11:**
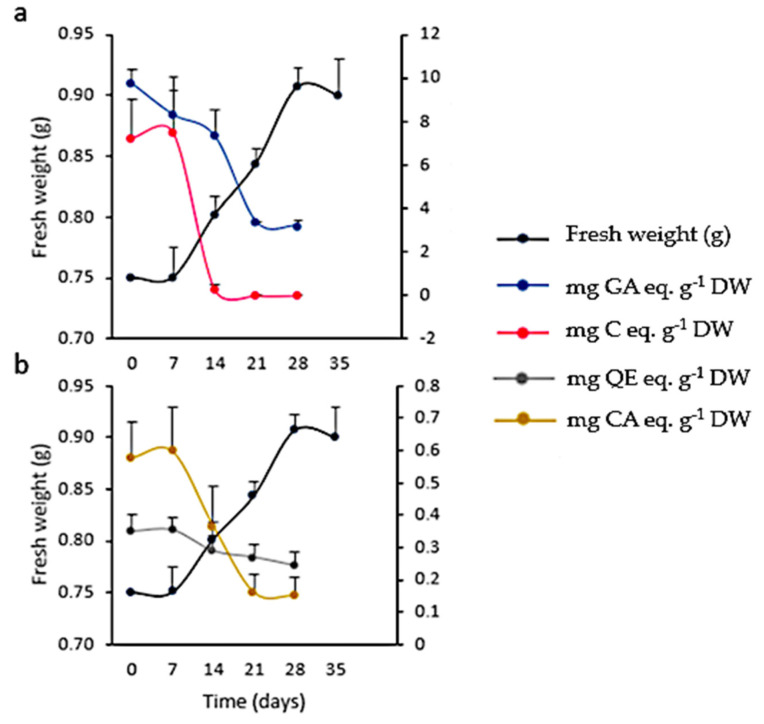
Growth and polyphenolic content in *Ligaria cuneifolia* cell suspension cultures growing in CWM with 2.50 µM NAA and 9.20 µM KIN as PGR. Inoculum size: 25 mg mL^−1^, photoperiod: 16 h, time of culture: 35 days (**a**,**b**). (**a**) Total phenolics (mg gallic acid eq·g^−1^ DW) and proanthocyanidins content (mg catechin eq·g^−1^ DW); (**b**) total flavonoids (mg quercetin eq·g^−1^ DW) and hydroxycinnamic acids content (mg chlorogenic acid eq·g^−1^ DW).

**Figure 12:**
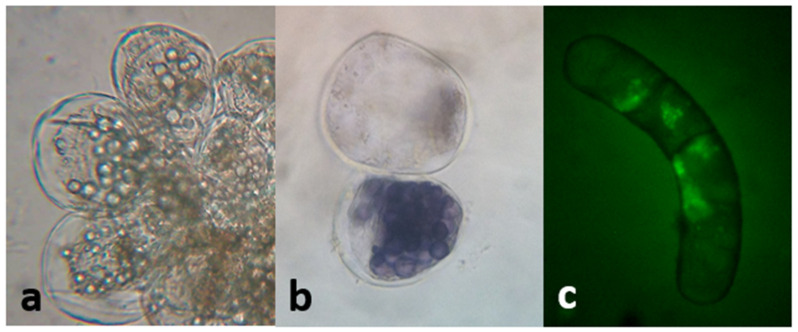
Cell suspension cultures of *L. cuneifolia* observed under the bright-field microscope. (**a**) Cell aggregates (40×); note the presence of intracellular starch granules; (**b**) cells observed under the bright-field microscope; positive Lugol’s test confirmed the presence of starch (40×); (**c**) fluorescent viable cell by the transformation of DAF into fluorescein (observation under UV light, 40×); a and b had the ability to exclude Evans blue at the plasma membrane and maintain their natural color.

A low inoculum size (3 mg mL^−1^) was not effective in inducing growth. On the other hand, with a higher inoculum size (25 mg mL^−1^) the growth curve from cell suspensions was sigmoidal with a lag phase of 7 days, followed by an exponential phase that extended up to the 28th day, when the culture entered the stationary phase (Figure 11). The kinetic parameters were GI = 1.07, µ = 0.01 d^−1^, and d_t_ = 66.89 d, with a biomass yield of 30.33 ± 0.67 mg mL^−1^. Microscopic analysis of the suspensions showed the presence of single or grouped cells forming aggregates. Cells had different morphologies, from spherical to oblong. The presence of starch was also observed (positive Lugol’s test and confirmed with polarized light). Evans blue and FDA tests proved that all cultures were viable (Figure 12). Mono-dimensional TLC showed a profile similar to that of calli and wild plant extracts but at a lower intensity (Figure 7b). The concentration of TPC, HCA, and PA was higher in the first week of culture and fell abruptly as the culture entered the exponential phase of growth. On the other hand, the low FL content fell smoothly from the 7th day of culture (from 0.35 ± 0.04 mg QE eq·g^−1^ DW to 0.24 ± 0.04 mg QE eq·g^−1^ DW; Figure 11). As was observed in calli, cell suspension cultures grew poorly under the conditions tested and did not produce a significant amount of polyphenolic compounds.

### 2.3. Antioxidant Activity of Wild Plants and In Vitro Cultures

**Figure 13:**
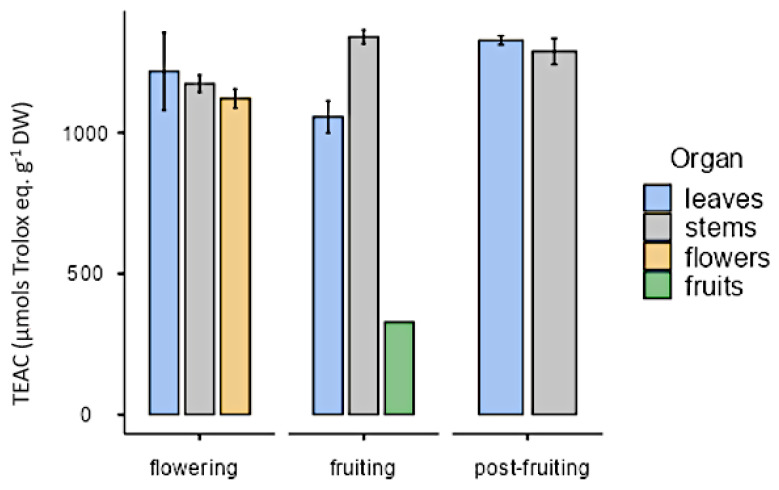
Antioxidant activity expressed as trolox equivalent antioxidant capacity (µmol trolox eq·g^−1^ DW), in organs from *L. cuneifolia* harvested in different phenological stages.

**Figure 14:**
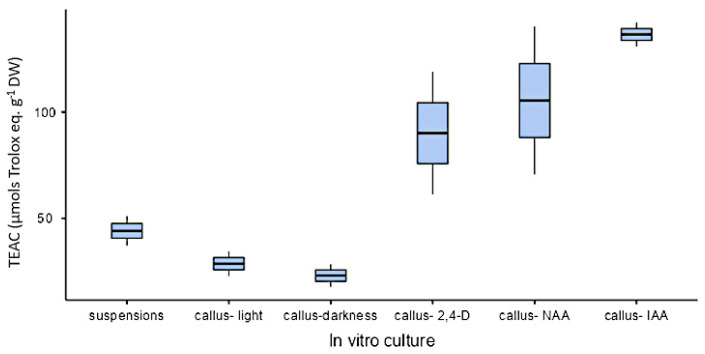
Antioxidant activity expressed as trolox equivalent antioxidant capacity (µmol trolox eq·g^−1^ DW), from in vitro cultures of *L. cuneifolia* growing in CWM under different conditions: suspension cultures in darkness with 2.50 µM NAA and 9.20 µM KIN; calli growing under a 16 h photoperiod or darkness with 2.50 µM NAA and 9.20 µM KIN; or calli growing under a 16 h photoperiod with IAA, NAA or 2,4-D at 2.5 µM. For calli culture 6.5 g L^−1^ of plant agar was added to CWM.

All organs at the phenological stages tested showed similar antioxidant activity (approximately 1000 µmol trolox eq·g^−1^ DW) except in the case of fruits that showed a lower value (328 µmol trolox eq·g^−1^ DW; Figure 13). As for in vitro cultures, both calli and cell suspension cultures showed antioxidant activity, but it was lower than in plants. On the other hand, antioxidant activity was higher under illumination (28.6 ± 8.21 µmol trolox eq·g^−1^ DW) than in darkness (23 ± 7.54 µmol trolox eq·g^−1^ DW) with the PGR ratio 2.50 µM NAA and 9.20 µM KIN. In calli cultures growing with auxins, the highest value was obtained in CWM with 2.5 µM IAA as PGR (137 ± 7.92 µmol trolox eq·g^−1^ DW). As for cell suspension cultures (25 mg mL^−1^ inoculum size, CWM with 2.50 µM NAA and 9.20 µM KIN), their antioxidant activity was 44.1 ± 9.84 µmol trolox eq·g^−1^ DW (Figure 14).

### 2.4. Mutagenicity and Antimutagenicity Assays of Wild Plants and In Vitro Culture Extracts

#### 2.4.1. Mutagenicity Assay

As can be seen in Table 6 and Table 7, none of the extracts or dilutions assayed showed mutagenic activity on *S. typhimurium* strains TA 98 and TA 100, with or without metabolic activation (RC values less than 2 indicate absence of mutagenicity). The results of the positive controls (not shown in the tables and not included in the CR calculations) for both strains gave values of CFU (colony forming units) greater than 1000 without metabolic activation, while when metabolic activation was performed, CFU values were 704.7 ± 55 for TA 100 and 919.67 ± 43.02 for TA 98.

#### 2.4.2. Antimutagenicity Assay

Once the assumption of homogeneity of variances was confirmed by the Bartlett test (*p* > 0.05), the results were compared using the Kruskal–Wallis test, and no significant differences were observed (*p* > 0.05) between the positive controls and the extracts tested. This indicates that the extracts obtained from wild specimens of *L. cuneifolia* did not show an antimutagenic effect.

## 3. Discussion

Phenolic compounds are redox-active species widely distributed in the plant kingdom. Among them, we focused our analysis on flavonoids (FL), hydroxycinnamic acids (HCA), and proanthocyanidins (PA) that, besides lectins, betulin, and betulinic acid, were the major components found in *L. cuneifolia* and considered responsible for their pharmacological activities [24]. Although phenolic compounds are synthesized in all parts of the plant, their content varies during plant growth and development. The content of phenolic compounds may vary depending on biotic (bacteria, fungi, parasites, predators) and abiotic (water, light, salts, chemicals, temperature, humidity, geographical variations, etc.) factors, the growth stage, and the part of the plant [25,26]. In the case of hemiparasites, another variable is added: the host. In this study, specimens were collected in Villa de Merlo (32°21′22.5″ S, 65°00′20.5″ W, 796 m.a.s.l.), in the province of San Luis. This location belongs to the Cuyo geographical region, an arid or semiarid climate with an average annual precipitation of about 100 to 500 mm and a pronounced temperature range from extremely hot temperatures during the day, followed by cold nights. From a phytogeographical perspective, this zone is located in the Neotropical region, specifically the *Chaqueño* domain with polymorphic vegetation and varied weather, in which the continental type is predominant with moderate to scarce rainfall, mild winters and warm summers [27]. Our research was focused on a single host, *Vachellia caven* (Mol.) Mol (Fabaceae), a common host of *L. cuneifolia*. Previous reports from our group also evaluated specimens from Barreal (31°38′00″ S, 69°28′00″ W, 1478 m.a.s.l.), in the province of San Juan, which is also part of the Cuyo geographical region, the Neotropical phytogeographical region and the *Chaqueño* domain. In that study, samples growing on *Prosopis chilensis* (Molina) Stuntz, *Prosopis flexuosa* D.C., and *Geoffroea decorticans* (Gillies ex Hook. & Arn.) Burkart, also from the Fabaceae family, were collected during the post-bloom stage. For comparative purposes, specimens from the Catamarca province (Belen, Puerta de San Jose, 27°33′0″ S, 67°1′0″ W, 1450 m.a.s.l.) developing on different hosts (*Olea europaea* L. Oleaceae, *Bulnesia retama* (Gillies ex Hook. & Arn.) Griseb. (Zygophyllaceae), *Geoffroea decorticans* (Gillies ex Hook. & Arn.) Burkart, and *Prosopis flexuosa* D.C.) were also included in our prior study [15]. This area belongs to the Northwest region, namely the Puna and is dry with a great temperature oscillation and mostly cold with subzero temperatures at night. It has been suggested that the phytochemical profile of mistletoes depends on the host of this parasitic plant [26]. However, the analysis of these combined findings strongly suggests that, from a qualitative perspective, *L. cuneifolia* FL fingerprint is highly conserved among geographical regions, climate conditions, and host families.

With regard to polyphenolic content, stems and leaves displayed the highest values, especially in the fruiting and post-fruiting stage. From a climatological point of view, temperature variation is not significant but the average precipitation is at its peak, so this period could be considered the “humid season.” This particular set of conditions favors plant metabolism, which results in a polyphenolic increase. Aerial parts are largely exposed to environmental conditions and different stressors, so a rapid turnover is generally observed. HCAs are prevalent in leaves, followed by stems, and are not significantly affected by the phenological stage. Polyphenols, and particularly FL, are secondary or “specialized” metabolites that provide protection and are involved in defense mechanisms. In terms of concentration, leaves showed the highest levels, especially at the post-fruiting stage where catechin is the predominant compound. This is consistent with the role of tannins as defense compounds. QE-3-*O*-glycosides and galloyl glycosides were also present at lower values. Our results are in agreement with those found in *V. album*, in which leaf extracts showed higher concentrations of total phenolics and flavonoids compared to fruit and seed extracts [28]. Similarities in polyphenolic profile but quantitative differences among growing seasons were also reported in *C. palirus* leaves. Several authors attributed the increase in phenolics content to higher intensity of solar radiation [29,30,31]. There has also been reported an increased expression of genes encoding phenylalanine ammonium lyase, chalcone synthase, and flavanone-3-β-hydroxylase in leaves of *Vaccinium myrtillus* exposed to sunlight [32]. These results correlate with those found in *L. cuneifolia* where the post-fruiting stage showed the highest polyphenolic values, coinciding with the summer season when solar radiation reaches its maximum. HCA were higher in leaves, and were not affected by the phenological stage. These results correlate with those found in *Geoffroea decorticans* extracts, in which leaves showed higher levels of HCA derivatives when compared to stems [33]. Chapel [16], working with *Calluna vulgaris* (L.) Hull, reported that the higher amounts of polyphenolic compounds, including HCA, were found in stems and leaves at all phenological stages except during flowering, while no significant differences in PA content were observed among different organs and phenological stages. As in *L. cuneifolia*, PA were also found in adult plants of the Mexican mistletoes *Phoradendron bollanum* and *Viscum album* subsp. *austriacum* [34]. HPLC results were consistent with a previous spectrophotometric analysis; leaves and stems showed the highest levels of total polyphenolics, especially in the post-fruiting stage, where catechin was the predominant compound. QE-3-*O*-glycosides and galloyl glycosides were also present but at lower values.

A selective spatial distribution was also seen in histochemical analysis, where flavonols and PA were located near the leaf surface, mostly in the epidermis and first parenchyma layers, which is consistent with the results reported in other plant species. Positive results were obtained in the reaction with vanillin/HCl, in *Microlaena stipoides*, *Eurycoma longifolia,* and *Themeda triandra,* which suggests the presence of condensed tannins and their precursors. The resulting pink coloration could indicate the existence of flavan-4-ols that produce anthocyanidins in the presence of concentrated HCl [35]. Ellis [36] found tannin-like compounds in the epidermal cells of the leaves of 39 genera and 101 species of South African grasses after histochemical analysis. On the other hand, FL were detected by histochemistry and confirmed by TLC, HPLC, UV/Vis spectroscopy, and mass spectrometry analysis, in *Arabidopsis thaliana* seedlings in three main zones: the cotyledonary node, the hypocotyl, and the root apical end [37], which is in agreement with our results with *L. cuneifolia* embryos.

As far as we know, there are few reports about the establishment of in vitro cultures from hemiparasitic plants for plant secondary metabolite production, e.g., chlorogenic acid production by *Viscum album* calli [38]. Most of the literature refers to the establishment of in vitro cultures from hemiparasite species, and particularly in the Loranthaceae family, to induce organogenesis [17,39,40], while phytochemical analysis was only performed in plants [17,41,42]. To our knowledge, this is the first report on species from the Loranthaceae family aiming at establishing in vitro culture conditions to produce polyphenolic compounds, particularly FL, HCA, and PA. Our results showed that *L. cuneifolia* calli presented a similar pattern of polyphenolic compounds as the adult plant regardless of the tested conditions. Illumination (16 h photoperiod) seemed to be a fundamental requisite for long-term maintenance and to polyphenolic production of calli cultures in *L. cuneifolia*. The positive effect of light on polyphenolic compounds accumulation could be related to the induction of some enzymes that participate in their biosynthetic pathway [43]. Shipilova [44], working with tea-plant calli, reported the increased PAL activity in those growing under a 16 h photoperiod, with the consequent increase in flavan concentration. Several authors have also reported the positive effect of light on growth and polyphenolic compound production by in vitro cell cultures, such as López-Laredo [45] working with *Tecoma stans* and Kumar [46] working with *Basella rubra.*

Considering the modest performance of calli in the previous assays and as PGRs are culture media components with utmost relevance in in vitro culture growth and biosynthetic capacity, we tested the influence on growth and polyphenolic content of calli growing on media with different auxins (IAA, NAA, and 2,4-D) at three concentrations (2.5, 5.0, and 10.0 µM). IAA (2.5 μM) appeared as the most favorable plant growth regulator for producing polyphenolic compounds. However, their amounts were low in comparison with those from organs of wild plants, except for PA. This group of polyphenols is rarely studied for in vitro production by plant cells [47,48,49].

As for cell suspension cultures, the presence of starch in cells was previously reported for calli cultures of Loranthaceae, as well as in suspension cultures of other plant species [50,51,52]. According to Fowler [53], sucrose administered to the culture medium is not only oxidized but also converted into starch as storage. However, it has been seen that, if further limitations of carbon source occur, this starch will not necessarily be used by cell cultures. Regarding growth kinetics, the best biomass yield was achieved when cultures were started from high-density inoculum. That result correlates with other reports. According to Torres [54], the use of low inoculum densities leads to a lengthening of the lag and exponential phases during growth. For each clone/culture medium there is a critical initial inoculum density, below which the culture will not grow. Mustafa [55] defined a general protocol for the establishment of cell suspensions, in which the following inoculum densities were determined: low (40–60 g PF L^−1^), medium (100–160 g PF L^−1^), and high (>200 g PF L^−1^). Álvarez [56] reached the highest biomass values in suspensions of *S. elaeagnifolium* starting from 20% (*v*/*v*) inoculum density (GI = 4) in MS medium with 50 µM NAA and 0.25 µM KIN. In the case of *Tilia americana*, batch suspensions (GI = 4.81 ± 0.88, d_t_ = 6.603 ± 0.78 d and μ = 0.107 ± 0.011 d^−1^) were started from 6% (*v*/*v*) inoculum density in MS medium supplemented with 2 mg L^−1^ 2,4-D and 0.5 mg L^−1^ KIN [57]. In *Capsicum baccatum*, the maximum GI (3.11) was achieved when using modified MS with the addition of 2,4-D (1.14 mg L^−1^) and BAP (0.23 mg L^−1^), with 12.5 g L^−1^ inoculum size [58]. Biomass doubling time varies with the species and culture conditions; for example, it was 60 h for *Acer pseudoplatanus*, 48 h for *N. tabacum*, 36 h for *Rosa* sp., and 24 h for *Phaseolus vulgaris*, among others [59]. It is noticeable that *L. cuneifolia* suspension cultures, under the conditions tested here, showed limited growth when compared with those reported for other plant species. It is necessary to continue optimizing other variables, such as the carbon source, the base culture medium, or the addition of other combinations of growth regulators to achieve better biomass yields for its subsequent scaling up to the bioreactor.

Regarding antioxidant activity, we found higher values in wild plant extracts, where polyphenol contents were also higher when compared to in vitro cultures. These in vitro results correlate with the in vivo and ex vivo experimental designs previously performed in *L. cuneifolia* [11]. In accordance with our findings, a linear correlation between the total phenolic level and antioxidant properties was described in *V. album*, which was attributed to the presence of phenolic compounds in earlier studies [28,60].

The Ames test is used to evaluate the mutagenic activity of a given chemical. In addition, it can be adapted for the detection of “protectants” or substances that decrease mutagenic action. We did not detect mutagenic or anti-mutagenic activity in any case.

## 4. Materials and Methods

### 4.1. Dynamics of Polyphenols in Different Organs and Phenological Stages of Wild Plants

#### 4.1.1. Plant Material

Plant material of *L. cuneifolia* (2–4 years old) growing on *Vachellia caven* (Mol.) Mol. was collected from Villa de Merlo, San Luis (32°21′22.5″ S, 65°00′20.5″ W). A specimen was deposited, under the name BAF 9018, in the herbarium of the “Juan Aníbal Domínguez” Pharmacobotany Museum at the Faculty of Pharmacy and Biochemistry, University of Buenos Aires. In 2018, branches of healthy specimens of *L. cuneifolia* were cut during the flowering, fruiting, and post-fruiting seasons (four, six, and five specimens, respectively). Flowering corresponded to the months of March and April (mean temperature 19.3–14.8 °C, air humidity 75.8–81.3%, and rainfall 11.8–3.6 mm), fruiting corresponded to the months of November (mean temperature 20.5 °C, air humidity 65.9%, and rainfall 17.19 mm), and post-fruiting to December (mean temperature 22.1 °C, air humidity 63.8%, and rainfall 69.8 mm). Finally, they were dried at room temperature in a dark and aerated area for approximately one week and were stored in a desiccator until they were used for polyphenol extraction. For histochemical analysis, fresh material was used. For that, medium- to large-size branches of *L. cuneifolia*-containing fruits were cut and moistened paper towels were placed around the cutting site. These were placed in sealable plastic bags and transferred to Ciudad de Buenos Aires in camping refrigerators or polystyrene boxes containing cooling gels, to keep the material as fresh as possible until its arrival at the laboratory, where it was kept at 4 °C until processing.

#### 4.1.2. Polyphenol Extraction

Dry leaves, stems, and flowers from adult wild specimens were separately ground in a rotary knife grinder (IKA). In both cases, a three-stage extraction was performed, first with pure methanol, then with 80% (*v*/*v*) methanol, and finally with 50% (*v*/*v*) methanol. Each stage was performed at room temperature for 24 h. The supernatants were pooled and stored at −20 °C for further analysis.

#### 4.1.3. Spectrophotometric Analysis

- Total phenolics content

Total phenolic content was determined using a modified Makkar [61] procedure. Briefly, 100 µL of the sample (calli extracts or plant extracts) was mixed with 100 µL of Folin–Ciocalteu reagent and kept in the dark at room temperature for 3 min. Then, 100 µL of 0.3 N sodium carbonate was added to the mixture. The mixture was then further incubated in the dark at room temperature for 30 min. The procedure was performed in 96-well microplates (Nunc™). The absorbance of the complex was measured at 765 nm using a microplate reader (µQuant, Biotek, VT, USA) and then compared to a standard curve prepared with various concentrations of gallic acid (100 µg mL^−1^ stock solution). The results were expressed in mg GA eq·g^−1^ DW. All experiments were performed in triplicate.

- Total flavonoid content

Total flavonoid content was determined using a modified Rafi [62] protocol. For calli, 50 µL of extract, 20 µL methanol, 10 µL of 10% (*w*/*v*) aluminum trichloride (AlCl_3_), 10 µL of 1 M potassium acetate, and 120 µL of distilled water were added to each well of a 96-well plate. For plant extracts, 25 µL of extract, 45 µL methanol, 10 µL of 10% (*w*/*v*) AlCl_3_, 10 µL of 1 M potassium acetate, and 120 µL of distilled water were added to each well of a 96-well plate. Then, the mixture was homogenized and incubated for about 30 min in the dark. The absorbance of the solution was measured at a wavelength of 415 nm in a microplate reader (µQuant, Biotek, VT, USA). Total flavonoid content was calculated with a QE calibration curve (stock solution 1 mg QE mL^−1^ in methanol) and the results were expressed as mg QE eq·g^−1^ DW. All experiments were performed in triplicate.

- Hydroxycinnamic acid content

HCA content was determined using a modified Ricco [63] procedure. Briefly, 15 µL of samples and 285 µL of ethanol were added to each well of a 96-well plate. Absorbance was measured at 328 nm in a microplate reader (µQuant, Biotek, VT, USA) and then compared to a calibration curve prepared with various concentrations of chlorogenic acid (1 mg mL^−1^ in absolute ethanol stock solution). The results were expressed as mg chlorogenic acid eq·g^−1^ DW. All experiments were performed in triplicate.

- Proanthocyanidin content

PA content was determined by a modified Horszwald and Andlauer [64] method. Briefly, 25 µL of samples mixed with 150 µL 4% (*w*/*v*) vanillin and 75 µL 32% (*v*/*v*) hydrochloric acid were added to each well of a 96-well plate. Then, the mixture was homogenized and incubated for about 15 min in the dark. Absorbance was measured at 500 nm in a microplate reader (µQuant, Biotek, VT, USA). Total PA content was calculated with a calibration curve prepared with various concentrations of (+)-catechin. Results were expressed as mg catechin eq·g^−1^ DW.

Statistical analysis: ANOVA and when corresponding a post hoc Tukey’s analysis were performed. Jamovi [65] and R [66] statistical software were used; *p* < 0.05 were considered significant.

#### 4.1.4. Thin-Layer Chromatography (TLC) Analysis

The qualitative analysis of polyphenols by thin-layer chromatography (TLC) was performed using Silica Gel 60 (Merck) as stationary phase and ethyl acetate:formic acid:acetic acid:water (100:11:11:23) as mobile phase. Flavonoids and HCA derivatives were revealed with a 1% (*v*/*v*) methanolic solution of the NP reagent and PA with a 5% (*w*/*v*) vanillin:HCl ethanolic solution [67].

#### 4.1.5. HPLC-UV Analysis

Considering the results obtained by spectrophotometric methods, extracts from calli, cell suspensions, and adult specimens were selected for HPLC-UV analysis [11]. Each analyte was compared among organs of the same phenological stage or PGR. Mean values followed by an asterisk and different letters represent statistically significant differences (*p* < 0.05, Tukey’s multiple comparison test).

#### 4.1.6. Histochemical Analysis

Cross sections of leaves, primary stems, and embryos were made with a sliding microtome. The sections were stained with 1% AEDBE methanolic solution for the detection of flavonoids and HCA derivatives, or with 5% vanillin:hydrochloric acid for the detection of PA [67]. All sections were observed under bright-field and fluorescence light microscopes.

### 4.2. In Vitro Culture Initiation, Growth Kinetics, and Polyphenolic Content under Different Culture Conditions

#### 4.2.1. Light Influence on Calli Induction, Growth, and Polyphenolic Content

Plant material for in vitro cultures was collected, preserved, and shipped to the lab as described in Section 4.1.1 (histochemical analysis). Fresh and mature fruits were disinfected as previously described [19]. Then, approximately 70 embryos were transferred to glass tubes (one per tube) containing control White modified medium (CWM = White medium with 500 mg L^−1^ casein hydrolysate, 100 mg L^−1^ myo-inositol, B5 vitamins, and 4% (*w*/*v*) sucrose) supplemented with 2.50 µM NAA, 9.20 µM KIN, and 6.5 g L^−1^ of agar (micropropagation grade); pH was adjusted to 5.6–5.8. One half of the glass tubes were cultured in a chamber with a 16/8 h (light/darkness) photoperiod given by fluorescent lamps (Narva T8 LT 18 W/760-010 daylight, Germany; irradiance 13.5 µmol m^−2^ s^−1^) and the other half in a chamber in darkness. Cultures were maintained at 24 ± 2 °C. After 60 days, the frequency of calli production (%) was estimated. Then, the established calli were transferred to identical fresh media and samples were taken once a week for 16 weeks to determine fresh weight (FW) and dry weight (DW) and for polyphenol qualitative and quantitative analysis as described in Section 4.1.2, Section 4.1.3, Section 4.1.4 and Section 4.1.5. Calli were oven dried at 40 °C until constant weight. Then, extraction was performed as described for wild plant specimens (Section 4.1.2). Experiments were made in duplicate and statistically analyzed by chi-square and Fisher’s tests with Jamovi software [65].

#### 4.2.2. Influence of PGRs and Inoculum Size on Growth Kinetics and Polyphenolic Content

Calli were transferred to glass tubes containing CWM with the addition of 1-naphthalene acetic acid (NAA), 3-indole acetic acid (IAA), or 2,4-dichloro phenoxy acetic acid (2,4-D) at 2.5, 5.0 or 10.0 µM. After four weeks of adaptation to the new media, the growth curve was initiated in the identical fresh medium using “low” inocula (100 to 250 mg FW) or “high” inocula sizes (250 to 500 mg FW; *n* = 29 for each treatment). Cultures were maintained in a growing chamber at 24 ± 2 °C, under a 16 h light photoperiod using fluorescent daylight lamps as previously detailed. Samples were taken every seven days during 8 weeks to measure FW and DW and for polyphenol qualitative and quantitative analysis as described in Section 4.1.3, Section 4.1.4 and Section 4.1.5. Calli were oven dried at 40 °C until constant weight. Then, extraction was performed as described for wild plant specimens (Section 4.1.2). Growth index (GI), specific growth rate (µ), and doubling time (*d_t_*) were calculated according to Equations (1)–(3), respectively. Statistical analysis was performed by ANOVA once the assumptions were confirmed. In case of significance in ANOVA, a Tuckey’s post hoc test was carried out. If lack of homoscedasticity was detected, a Welch’s heteroscedastic F-test [68,69] was performed, with a Games–Howell post hoc test when necessary [70,71]. Jamovi and R Studio software were used [65,66].

Equation (1): formula for calculating the growth index (*GI*). *x*: biomass at time t; *x*_0_: initial biomass

(1) $$GI=\frac{x}{x_{0}}$$

Equation (2): formula for calculating the specific growth rate (µ). *x*: biomass at time *t*; *x*_0_: initial biomass; *t*: final time; *t*_0_: initial time

(2) $$µ=\frac{\ln\left(\frac{x}{x_{0}}\right)}{t-t_{0}}$$

Equation (3): formula for calculating the doubling time (*d_t_*). µ: specific growth rate

(3) $$d_{t}=\frac{ln_{2}}{µ}$$

#### 4.2.3. Establishment of Cell Suspension Cultures

Calli growing in semi-solid CWM with 2.50 µM NAA and 9.20 µM KIN as PGRs were transferred to 150 mL Erlenmeyer flasks containing 30 mL of the same culture medium but without agar. Two inoculum sizes were tested, 100 mg/flask (low) and 750 mg/flask (high). The flasks were transferred to an orbital shaker at 110 rpm, 24 ± 2 °C, and maintained in the dark. Samples were taken on days 7, 14, 21, and 28 to determine viability (Evans blue and fluorescein diacetate tests [72]), FW, and DW and for polyphenol qualitative and quantitative analysis as described in Section 4.1.3, Section 4.1.4 and Section 4.1.5. Cell suspensions were filtered and dried at 40 °C until constant weight. Then, extraction was performed as described for wild plant specimens (Section 4.1.2).

### 4.3. Antioxidant Activity of Wild Plants and In Vitro Cultures Extracts

Wild plant and *in vitro* culture extracts were obtained as described in Section 4.1.2. Antioxidant activity was determined according to the method of Cheng [73] by using 2,2-diphenyl-1-picrylhydrazyl (DPPH; a radical organic compound with two absorption peaks: 340 and 515 nm) that reduces its absorption at 515 nm in the presence of antioxidant compounds. Results were expressed as mg trolox eq·g^−1^ DW and descriptive statistical analysis was performed

### 4.4. Mutagenicity and Antimutagenicity Assays of Wild Plants and In Vitro Culture Extracts

#### 4.4.1. Mutagenicity Assay

The Ames test was carried out to evaluate the possible mutagenicity of the extracts of *L. cuneifolia* wild plants and in vitro cultures. *Salmonella typhimurium* TA98 and TA100 strains were used in order to detect frameshift mutations and base-pair exchanges, respectively [74]. Tests were carried out to confirm the phenotypic characteristics for resistance to ampicillin, the rfa mutation (permeability to crystal violet), and the uvrB mutation (sensitivity under UV light). The test was also carried out with and without the addition of S9 mix (metabolic activator) by means of the plate incorporation method. The test is considered positive when, at least, the number of spontaneous revertants is doubled. In tubes containing 2 mL of soft agar (at 45 °C) supplemented with L-histidine HCl-D-biotin (0.5 mM–0.5 mM), 50 µL of a culture incubated for 12 h with a density of 1 × 108 cells mL^−1^ of the selected control strains (TA98 or TA100), and 0.5 mL of S9 mix for the assay with metabolic activation or phosphate buffer (pH 7.4) in the assays without metabolic activation. Then, 50 µL of the dilution of the wild plant or calli extract was added. This mixture was gently homogenized with a vortex and plated with a minimal medium for its incubation at 37 °C for 48 h. Then, the revertant His+ colonies were counted. In all the tests, negative controls were included to know the rate of appearance of spontaneous revertant colonies. As positive controls, B1 aflatoxin (2 µg/plate) and 2-aminoanthracene (5 µg/plate) were used for the assays with metabolic activation in both strains, and sodium azide (5 µg/plate) and 2-aminofluorene (10 µg/plate) for assays without metabolic activation, in TA 100 and TA 98, respectively. Results were expressed as the reversion coefficient or C.R. = No. of revertant colonies per assayed plate/No. of revertant colonies per control-spontaneous-plate.

#### 4.4.2. Antimutagenicity Assay

A volume of 0.5 mL of S9 mix, 50 µL of B1 aflatoxin, and 50 µL of tester strain suspension was placed in each tube and brought to a final volume of 2 mL with water. 50 µL of water were added to the positive control, and 50 µL of wild plant extract to the unknown sample. A blank without aflatoxin was also performed in which 100 µL of water and an extract blank without B1 aflatoxin and with 50 µL of water were used. The results were statistically analyzed using the Bartlett test to determine the homogeneity of variances and then compared using the Kruskal–Wallis test.

### 4.5. Chemicals and Reagents

White medium [75], sucrose, casein hydrolysate and agar (plant tissue micropropagation culture grade) were from PhytoTechnology Laboratories (Lenexa, KS, USA). LiChrosolv^®^ Methanol was supplied by Merck (Darmstadt, Germany). Formic acid was purchased from Baker (New Jersey, USA). Ultrapure water was generated with a Barnstead Thermo Scientific™ (Waltham, MA, USA). Quercetin-3-rhamnoside (Q-3-*O*-Rh) was from Extrasynthese (Lyon, France); catechin (C), quercetin-3-*O*-glucoside (Q-3-*O*-G), quercetin-3-*O*-xyloside (Q-3-*O*-X), quercetin-3-*O*-arabinofuranoside (Q-3-*O*-AF), quercetin-3-*O*-arabinopyranoside (Q-3-*O*AP), and chlorogenic acid (CA) were from Sigma (St. Louis, MO, USA). The other chemicals, standards, and solvents were purchased from Sigma-Aldrich (Saint Louis, MO, USA).

## 5. Conclusions

Regarding wild plants, the post-fruiting stage proved to be the best time of harvesting to obtain the maximum amounts of polyphenolic compounds. Further experiments will be performed to continue analyzing the influence of different host plants on the polyphenolic profile. *L. cuneifolia* appeared to be a very challenging species to be introduced in in vitro culture. Different PGR ratios, light conditions, and inoculum sizes were tested, and only a slight improvement in growth and polyphenolic content was achieved. We established that, among the conditions tested, calli grew and produced FL, HCA, and PA in CWM with 2.5 µM IAA and 16 h photoperiod, but at low levels compared to wild plants. As for cell suspension cultures, the conditions tested were not successful either for growth or for polyphenolic production. Other media components such as carbon and nitrogen source and PGR ratios must be assayed to establish the optimal conditions to obtain cultures with better growth. Then, other strategies such as elicitation or two-step cultures (culturing cell suspensions in a medium that promotes growth and then transferring them to a medium that favors polyphenolic production) could be examined to obtain improved yields. No mutagenic or antimutagenic activity was detected in wild plants or in in vitro culture methanolic extracts.

## Figures and Tables

**Table 1:** Concentration of total phenolics (mg gallic acid eq·g^−1^ DW) according to the phenological stage and organ from *Ligaria cuneifolia*. Results were expressed as mean ± SD.

Phenological Stage	Organ	Total Phenolics (mg Gallic Acid eq·g^−1^ DW) ^1^
Flowering	Flowers	179 ± 22.0
	Leaves	168 ± 1.9
	Stems	183 ± 13.9
Fruiting	Fruits	81.7 ± 0.00
	Leaves	170 ± 4.86
	Stems	209 ± 13.7
Post-fruiting	Leaves	207 ± 17.5
	Stems	214 ± 12.1

^1^ Two-way ANOVA results for phenological stage: F (2.24) = 15.52, *p* < 0.001; and for organ: F (1.24) = 15.94, *p* < 0.001.

**Table 2:** Tukey’s post hoc test comparing *Ligaria cuneifolia* leaves and stems in different phenological stages for the variable total phenolics. df: degrees of freedom; SE: standard error.

Organ	Phenological Stage	Organ	Phenological Stage	Mean Difference	SE	df	t	*p*_tukey_
Leaves	Flowering	Leaves	Fruiting	−2.45	8.74	24	−0.28	1
		Leaves	Post-fruiting	−39.58	9.09	24	−4.355	0.003 ^1^
		Stems	Flowering	−15.29	9.58	24	−1.596	0.609
		Stems	Fruiting	−40.87	8.74	24	−4.674	0.001 ^1^
		Stems	Post-fruiting	−45.94	9.09	24	−5.055	< 0.001 ^1^
	Fruiting	Leaves	Post-fruiting	−37.13	8.20	24	−4.527	0.002 ^1^
		Stems	Flowering	−12.84	8.74	24	−1.468	0.686
		Stems	Fruiting	−38.42	7.82	24	−4.912	< 0.001 ^1^
		Stems	Post-fruiting	−43.49	8.20	24	−5.302	< 0.001 ^1^
	Post-fruiting	Stems	Flowering	24.29	9.09	24	2.673	0.118
		Stems	Fruiting	−1.29	8.20	24	−0.157	1
		Stems	Post-fruiting	−6.36	8.57	24	−0.742	0.974
Stems	Flowering	Stems	Fruiting	−25.58	8.74	24	−2.926	0.071
		Stems	Post-fruiting	−30.65	9.09	24	−3.373	0.027 ^1^
	Fruiting	Stems	Post-fruiting	−5.07	8.20	24	−0.618	0.989

^1^ Significant differences (*p* < 0.05).

**Table 3:** Concentration of total hydroxycinnamic acids (mg chlorogenic acid eq·g^−1^ DW) according to the phenological stage and organ from *Ligaria cuneifolia*. Results were expressed as mean ± SD.

Phenological Stage ^1^	Organ ^1^	Hydroxycinnamic Acids (mg Chlorogenic Acid eq·g^−1^ DW)
Flowering	Flowers	2.05 ± 0.40
	Leaves	3.02 ± 0.62
	Stems	2.50 ± 0.26
Fruiting	Fruits	2.77 ± 0.01
	Leaves	2.42 ± 0.28
	Stems	2.24 ± 0.41
Post-fruiting	Leaves	3.06 ± 0.69
	Stems	2.40 ± 0.49

^1^ Two-way ANOVA results for phenological stage: F (1.24) = 6.601, *p* = 0.017 and for organ F (2.24) = 2.700, *p* = 0.088.

**Table 4:** Total proanthocyanidin content in different *Ligaria cuneifolia* organs and phenological stages. Results were expressed as mean ± SE.

Phenological Stage ^1^	Organ ^1^	Proanthocyanidins (mg Catechin eq·g^−1^ DW)
Flowering	Flowers	32.5 ± 9.60
	Leaves	32.4 ± 7.25
	Stems	32.4 ± 5.99
Fruiting	Fruits	23.9 ± 5.0
	Leaves	29.0 ± 12.0
	Stems	32.7 ± 6.87
Post-fruiting	Leaves	35.3 ± 5.79
	Stems	34.6 ± 8.59

^1^ Two-way ANOVA results for phenological stage: F (1.24) = 0.103, *p* = 0.751 and for organ F (2.24) = 0.656, *p* = 0.528.

**Table 5:** Concentration of polyphenols detected by HPLC-UV of methanolic extracts from different organs (leaves, stems, flowers, and fruits) harvested in different phenological stages (flowering, fruiting, and post-fruiting), from calli grown with different PGRs (2,4-D, NAA and IAA all at 2.5 µM) and grown under different illumination conditions (16 h photoperiod and darkness) in CWM with 2.50 µM NAA and 9.20 µM KIN. C (Catechin). Q-3-*O*-gluc (Q-3-*O*-Glucoside). Q-3-*O*-xyl (Q-3-*O*-xyloside). Q-3-*O*-AP (Q-3-*O*-arabinopyranoside). Q-3-*O*-AF (Q-3-*O*-arabinofuranoside). Q-3-*O*-2-gal-AF (Q-3-*O*-2-galloyl-arabinofuranoside). Q-3-*O*-R (Q-3-*O*-rhamnoside). Q-3-*O*-2-gal-R (Q-3-*O*-2-galloyl-rhamnoside) and Q-3-*O*-3-gal-R (Q-3-*O*-3-galloyl-rhamnoside). ND: not detected. Values are expressed as mean ± SEM.

Phenological Stage/Treatment	Organ	Concentration (mg g^−1^ Dry Weight)									
		C	Q-3-O-gluc	Q-3-O-xil	Q-3-O-AP	Q-3-O-AF	Q-3-O-2-gal-AF	Q-3-O-R	Q-3-O-2-gal-R	Q-3-O-3-gal-R	Total
Flowering	Flowers	8.65 *^a^ ± 0.08	0.16 ± 0.02	0.30 *^a^ ± 0.02	0.35 ± 0.15	0.63 *^a^ ± 0.05	0.48 *^a^ ± 0.06	0.83 *^a^ ± 0.15	0.32 *^a^ ± 0.06	0.55 *^a^ ± 0.15	12.27 *^a^ ± 0.63
Flowering	Stems	7.43 ± 0.01	0.18 ± 0.02	0.24 *^b^ ± 0.04	0.33 ± 0.05	0.35 *^b^ ± 0.03	0.33 *^b^ ± 0.03	0.49 *^b^ ± 0.03	0.41 *^b^ ± 0.09	0.25 *^b^ ± 0.01	10.01 *^b^ ± 0.19
Flowering	Leaves	7.41 ± 0.31	0.32 *^a^ ± 0.18	0.56 *^c^ ± 0.01	0.66 *^a^ ± 0.08	1.01 *^c^ ± 0.13	0.82 *^c^ ± 0.06	1.42 *^c^ ± 0.58	0.73 *^c^ ± 0.03	0.74 *^c^ ± 0.10	13.71 *^c^ ± 0.69
Fruiting	Stems	6.96 *^a^ ± 0.84	0.18 ± 0.02	0.26 ± 0.06	0.28 *^a^ ± 0.08	0.42 *^a^ ± 0.12	0.37 *^a^ ± 0.03	0.67 *^a^ ± 0.29	0.50 *^a^ ± 0.06	0.47 *^a^ ± 0.05	10.11 *^a^ ± 1.15
Fruiting	Leaves	8.66 *^b^ ± 0.66	0.25 *^a^ ± 0.01	0.32 *^a^ ± 0.04	0.22 *^b^ ± 0.04	0.56 *^b^ ± 0.08	0.51 *^b^ ± 0.07	0.91 *^b^ ± 0.29	0.62 *^b^ ± 0.04	0.81 *^b^ ± 0.01	12.86 *^b^ ± 0.30
Fruiting	Fruit	0.86 *^c^ ± 0.02	0.20 ± 0.01	0.24 ± 0.02	0.50 *^c^ ± 0.02	0.72 *^c^ ± 0.01	0.72 *^c^ ± 0.01	0.40 *^c^ ± 0.02	0.16 *^c^ ± 0.01	0.52 *^c^ ± 0.02	4.32 *^c^ ± 0.04
Post-fruiting	Stems	13.81 ± 0.98	0.28 ± 0.01	0.57 ± 0.05	0.63 ± 0.03	0.99 ± 0.09	0.98 ± 0.12	1.02 ± 0.20	0.54 ± 0.01	1.62 ± 0.97	20.44 ± 0.36
Post-fruiting	Leaves	12.16 ± 0.95	0.24 ± 0.02	0.89 ± 0.15	0.79 ± 0.13	1.24 ± 0.08	1.27 ± 0.11	1.65 ± 0.03	0.53 ± 0.11	0.87 ± 0.13	19.64 ± 0.24
2.4-D 2.5 µM	Calli	0.99 *^a^ ± 0.51	0.08 *^a^ ± 0.01	0.08 *^a^ ± 0.02	0.15 *^a^ ± 0.01	0.19 ± 0.05	0.13 *^a^ ± 0.05	0.31 *^a^ ± 0.25	0.10 *^a^ ± 0.08	0.05 *^a^ ± 0.03	2.08 *^a^ ± 0.02
IAA 2.5 µM	Calli	1.97 *^b^ ± 0.31	0.11 *^b^ ± 0.01	0.12 *^b^ ± 0.04	0.27 *^b^ ± 0.07	0.21 *^a^ ± 0.05	0.10 *^b^ ± 0.04	0.37 *^b^ ± 0.29	0.05 *^b^ ± 0.01	0.07 *^b^ ± 0.03	3.27 *^b^ ± 0.77
NAA 2.5 µM	Calli	0.60 *^c^ ± 0.36	0.02 *^c^ ± 0.02	0.04 *^c^ ± 0.02	0.08 *^c^ ± 0.01	0.09 *^b^ ± 0.05	0.04 *^c^ ± 0.02	0.10 *^c^ ± 0.08	0.02 *^c^ ± 0.01	0.01 *^c^ ± 0.01	1.00 *^c^ ± 0.48
Darkness	Calli	0.07 ± 0.01	ND	ND	ND	0.09 ± 0.01	0.07 ± 0.03	0.07 ± 0.03	0.04 ± 0.01	0.07 ± 0.01	0.40 ± 0.02
Light	Calli	0.02 ± 0.01	0.03 ± 0.01	0.07 ± 0.03	0.23 ± 0.05	0.19 ± 0.09	0.08 ± 0.05	0.19 ± 0.08	0.03 ± 0.01	0.03 ± 0.01	0.87 ± 0.08

Each analyte was compared among organs of the same phenological stage or PGR. Mean values followed by an asterisk and different letters (a–c) represent statistically significant differences (*p* < 0.05, Tukey’s multiple comparison test).

**Table 6:** Results of the Ames test in TA 100 and TA 98 *S. typhimurium* strains with (+S9) and without (−S9) metabolic activation. A mixture of equal parts of leaf and stem methanolic extracts from specimens harvested in the post-fruiting season was tested. Results were expressed as the reversion coefficient (RC). SD: standard deviation.

	RC ± SD—Adult Specimen Extracts			
	TA 100 + S9	TA 100 − S9	T 98 + S9	TA 98 − S9
¼ diluted extract	0.95 ± 0.09	1.02 ± 0.05	1.01 ± 0.12	1.00 ± 0.05
½ diluted extract	0.97 ± 0.04	1.01 ± 0.03	0.99 ± 0.14	0.96 ± 0.09
Direct extract	0.84 ± 0.04	0.76 ± 0.17	0.78 ± 0.06	0.86 ± 0.18

**Table 7:** Results of the Ames test in TA 100 and TA 98 *S. typhimurium* strains with (+S9) and without (−S9) metabolic activation. Methanolic extracts from calli grown in White medium with 500 mg L^−1^ casein hydrolysate, 4% (*w*/*v*) sucrose, and 2.50 µM NAA were tested. Results were expressed as the reversion coefficient (RC). SD: standard deviation.

	RC ± SD—*In Vitro* Cultures Extracts			
	TA 100 + S9	TA 100 − S9	T 98 + S9	TA 98 − S9
¼ diluted extract	1.00 ± 0.07	1.02 ± 0.06	1.07 ± 0.04	1.04 ± 0.05
½ diluted extract	1.03 ± 0.03	1.03 ± 0.04	1.02 ± 0.12	1.01 ± 0.13
Direct extract	0.92 ± 0.07	0.94 ± 0.04	1.00 ± 0.03	0.81 ± 0.18

## Data Availability

The data presented in this study are available upon request from the corresponding author.
